# Risk factors for postoperative delirium in frail elderly patients undergoing on-pump cardiac surgery and development of a prediction model—a prospective observational study

**DOI:** 10.3389/fcvm.2024.1425621

**Published:** 2024-08-07

**Authors:** Yinyin Ding, Ju Gao, Yali Ge, Tianfeng Huang, Yang Zhang

**Affiliations:** Department of Anesthesiology, North Jiangsu people's Hospital Affiliated to Yangzhou University, Yangzhou, Jiangsu, China

**Keywords:** frailty, cardiopulmonary bypass, tissue oxygen saturation, cardiac surgery, postoperative delirium, near-infrared spectroscopy

## Abstract

**Background:**

To identify the risk factors for postoperative delirium (POD) after cardiac surgery in frail elderly patients and develop a receiver operating characteristic (ROC) prediction model to confirm the effectiveness.

**Methods:**

This was a prospective observational study, patients were assessed preoperatively according to the frailty index (FI) scale. Cerebral (SctO_2_) was assessed at different time points using near-infrared spectroscopy (NIRS). On the basis of the occurrence of POD within 7 days after surgery, patients were divided into POD and non-POD groups. Risk factors were analyzed using logistic regression analysis, while their predictive values were evaluated using the receiver operating characteristic curve analysis.

**Results:**

POD was significantly associated with frailty, lower preoperative MMSE scores, hyperlipidemia, diabetes, cerebrovascular disease, lower hemoglobin level, lower albumin level, longer operation time, longer CPB time, lower SctO_2_ at T5, and lower SctO_2baseline_ (*P* < 0.05). SrtO_2_ and SmtO_2_ did not differ significantly between groups. FI, preoperative MMSE score, and operation time as independent risk factors (*P* < 0.05). Significant predictive value was demonstrated in all 3 variables (*P* < 0.001; respectively). Among them, high sensitivity and specificity were observed with the FI (cut-off value 0.27, sensitivity 75%, specificity 73.5%) and operation time (cut-off value 237.5, sensitivity 62.5%, specificity 78.6%).

**Conclusions:**

The FI, preoperative MMSE score, and operation time were independent risk factors for POD in elderly patients after cardiac surgery, with high predictive value observed with the FI and operation time. Cerebral oxygen saturation was associated with POD but was not an independent risk factor.

**Clinical Trial Registration:**

Chinese Clinical Trail Registry, No: chictr2200056038.

## Introduction

Frailty is an age-related state of diminished physiological reserve and function that results in increased vulnerability to endogenous and exogenous stressors (1–3). With the increasing prevalence of cardiovascular diseases among elderly patients, the incidence of frailty has been increasing among cardiac surgical patients of these age groups (4–6). Limitations in withstanding surgical stressors (7) are associated with an increased risk of adverse health outcomes such as surgery-related complications, falls, disability, hospitalization, and mortality (8, 9).

Postoperative delirium (POD) represents an acute neurocognitive complication characterized by fluctuating levels of consciousness (10, 11). It mainly occurs within 2–3 days after surgery (12), and has been considered the most common surgical complication among elderly patients (13). Besides the negative impacts of POD on postoperative recovery, it often results in prolonged hospital stay, increased medical costs, as well as increased risk of patient morbidity and mortality (14). The incidence of POD varies across different types of surgeries. In China, the overall incidence of POD in hospitalized patients older than 65 years of age is 17.1% (15), with cardiac surgery having a 46% incidence of POD due to the intraoperative effects of extracorporeal circulation (16). The etiology of POD is multifactorial (17), and may involve both intrinsic and extrinsic factors such as age, cognitive dysfunction, comorbidities, visual and hearing status, and alcohol use, among others (18, 19).

The aim of the study is to explore the risk factors for POD in frail elderly patients undergoing on-pump cardiac surgery, to further improve the clinical prevention and treatment of POD among such patients.

## Methods

### Study design

This study was approved by the Institutional Committee for Medical Ethics (2022ky002), and has been registered on the Chinese Clinical Trial Registry (https://www.chictr.org.cn/ChiCTR2200056038).

Patients undergoing elective on-pump cardiac surgery at Northern Jiangsu People's Hospital (Yangzhou City, Jiangsu province, China) between November 2021 and May 2022 were prospectively enrolled. Inclusion criteria included age ≥65 years. Exclusion criteria included (1) hepatic or renal insufficiency; (2) not capable of self-care; (3) visual and hearing impairment; (4) history of severe neuropsychiatric illness; (5) recent sedatives or antidepressant use; (6) cerebrovascular diseases such as intracranial aneurysm, head and neck artery stenosis or occlusion, and vascular cognitive impairment, (7) hemorrhagic stroke or traumatic brain injury with sequelae, (8) inability to cooperate with study protocol, (9) preoperative Mini-Mental State Examination (MMSE) score <20, and (10) emergency surgeries.

### Perioperative management

Baseline frailty status was assessed using the FI scale. Patients were assessed preoperatively according to the FI, and frailty was defined as FI ≥0.25; pre-frailty was defined as FI 0.09–0.25. Cerebral oxygen saturation was monitored using the FORE-SIGHT oximeter (CASMED, Branford, CT, USA). Renal and brachioradialis muscle oxygen saturations were monitored using the INVOS5100C device (Medtronic, Minneapolis, MN).

### Study protocol

A single blinded anesthetist was involved in the study, and anesthetic management was not influenced by the oxygen saturation monitoring values. Induction was performed with a single injection of midazolam (0.1–0.5 mg/kg), etomidate (0.1–0.4 mg/kg), sufentanil (0.5 ug/kg), and cisatracurium (0.2 mg/kg). The pressure-controlled ventilation volume-guaranteed (PCV-VG) mode was used with the following parameters: fraction of inspired oxygen (FiO_2_), 0.6–0.8; tidal volume, 6–8 ml/kg (employing ideal body weight); PEEP, 5 cmH_2_O; and partial carbon dioxide pressure (PaCO_2_), 40 mmHg, as measured using an arterial blood gas analyzer (Gem 3500, Instrumentation Laboratory, Bedford, MA, USA). Maintenance was achieved with dexmedetomidine (0.2–0.5 ug/kg/h), cisatracurium (0.05–0.1 mg/kg/h), and sevoflurane (1%–2.5%). Sufentanil was administered as necessary throughout the procedure. Noradrenaline, dopamine, dobutamine, and nitroglycerin were used to maintain hemodynamic balance. MAP was maintained within 20% of baseline.

Extracorporeal circulation was performed using a heart-lung machine (MAQUET, Rastatt, Germany) and a membrane oxygenator (TERUMO Corporation, Tokyo, Japan). Heparin (300 U/kg) was administered to achieve an activated coagulation time (ACT) of ≥480 s. Antegrade hyperkalemic cardioplegia was instituted, and moderate hypothermia was applied (32–35°C). Perfusion flow rate (2.0–2.4 L/min/m^2^) was adjusted to maintain a perfusion pressure of 60–80 mmHg. Blood pH and hematocrit were maintained at 7.35–7.45 and 25%–30%, respectively. ACT was monitored dynamically, and heparin was added as necessary. Upon completion of the procedure, slow infusion of protamine was administered via the ascending aorta for neutralization.

The FI scale we used assessed 50 items, including mental status, self-care ability, motor coordination, adverse living habits, head and neck diseases, cardiovascular diseases, pulmonary diseases, abdominal diseases, cerebrovascular disease, hypertension, hyperlipidemia, diabetes, skin diseases, musculoskeletal diseases, sleep quality, physiological and mental illness, cognitive disorders, standing and walking posture, and history of falls. The patient FI was defined as the ratio of the number of items showing defects to the total number of assessed items. The total score was 1. Frailty, pre-frailty, and non-frailty were defined as FI ≥0.25, FI 0.09–0.25, and FI ≤0.08, respectively.

Baseline demographic and clinical data included sex, age, body mass index (BMI), noninvasive and invasive blood pressure, the American Society of Anesthesiologist (ASA) score, New York Heart Association (NYHA) classification, MMSE score, presence of comorbidities such as hypertension, diabetes, hyperlipidemia, cerebrovascular disease, intraoperative vasoactive drug doses, hemoglobin, white cell count, albumin, N-terminal pro-B-type natriuretic peptide, S100β calcium binding protein, left ventricular ejection fraction, left atrium diameter, left ventricle diameter, and surgery type.

Intraoperative data included doses of the vasoactive drugs, operation time, mechanical ventilation time, CPB time, aortic cross clamp time, and tissue oxygen saturation. Cerebral (SctO_2_), renal (SrtO_2_), and brachioradialis muscle oxygen saturation (SmtO_2_) were recorded at the following time points: 1 day pre-surgery (T0), 5 min post-admission (T1), pre-tracheal intubation (T2), 5 min post-tracheal intubation (T3), pre-CPB (T4), at aortic clamp (T5), at aortic clamp opening (T6), the end of CPB (T7), post-surgery (T8), followed by post-surgery day 1 (T9), 3 (T10), 5 (T11), and 7 (T12). The baseline (SctO_2 baseline_, SrtO_2 baseline_, SmtO_2 baseline_), lowest (SctO_2 lowest_, SrtO_2 lowest_, SmtO_2 lowest_), and greatest (SctO_2 decrease_, SrtO_2 decrease_, SmtO_2 decrease_) oxygen saturation values were recorded.

Postoperative data included complications, as well as cardiac intensive care unit (ICU) and hospital length of stay. The postoperative complications included postoperative pulmonary complications (pneumonia, atelectasis, pulmonary embolism, pulmonary insufficiency), postoperative nausea and vomiting, and sternal wound infections (both deep and superficial infections).

### Statistical analysis

All statistical analyses were performed using SPSS version 25. Normality of distribution was assessed using the Kolmogorov-Smirnov test. Normally distributed data are expressed as mean ± standard deviation (SD), with intra- and inter-group comparisons made using Student *t*-test, respectively. Non-normally distributed data were compared using Mann-Whitney *U*-test. Numerical data were compared using the chi-square test. Risk factors for POD were analyzed using logistic regression, and their predictive values were assessed using the receiver operating characteristic (ROC) curve analysis.

### Sample size estimation

The sample size was calculated with reference to the preliminary study and based on the sample size calculation formula. Since the probability of postoperative delirium was *p* = 0.6 for group A and *p* = 0.3 for group B, then for equal N in each group, *α* = 0.05, and (1-β) = 0.90, the required sample size was *N* = 112.

## Results

### Patient characteristics

**Figure 1:**
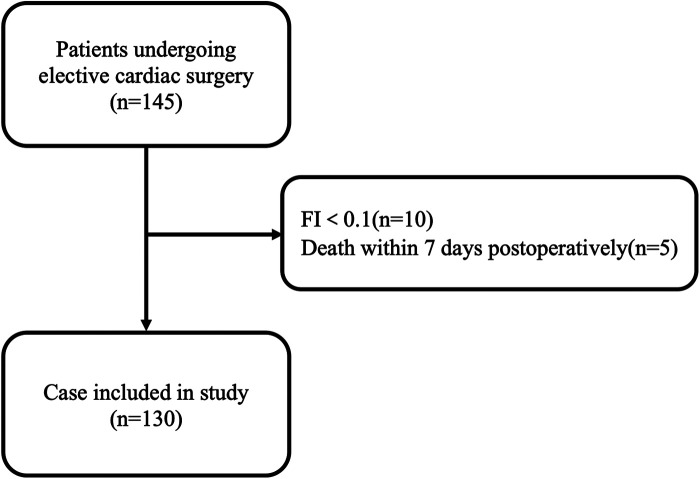
Flow diagram of the patient selection process. FI, frailty index scale.

A total of 145 patients were scheduled for on-pump cardiac surgery during the study period (Figure 1). Among them, 15 were excluded due to FI <0.1 (*n* = 10), and death within 7 days of surgery (*n* = 5). As such, 130 patients were eventually included for data analysis.

### Univariate logistic regression analysis results of potential clinical and demographic risk factors

A total of 32 patients (24.6%) demonstrated POD. The clinical data of the POD and Non-POD groups are summarized in Table 1. Based on univariate analysis, the FI, preoperative MMSE score, hyperlipidemia, diabetes mellitus, cerebrovascular disease, hemoglobin and albumin levels, operation time and CPB time were found as significant risk factors for POD (Table 1).

**Table 1:** Univariate logistic regression analysis results of potential clinical and demographic risk factors.

		Non-POD (*n* = 98)	POD (*n* = 32)	*P*-value
Age [year, (Q1, Q3)]		67 (64–72.3)	68 (60–74)	0.965
Sex, *n* (%)	M	60 (61.2%)	16 (50%)	0.237
	F	38 (38.8%)	16 (50%)	
ASA, *n* (%)	Ⅳ	95 (96.9%)	29 (90.6%)	0.143
	Ⅴ	3 (3.1%)	3 (9.4%)	
NYHA, *n* (%)	Ⅲ	32 (32.7%)	6 (18.8%)	0.125
	Ⅳ	66 (67.3%)	26 (81.3%)	
BMI [kg/m^2^,(x ± s)]		22.7 ± 3.5	22.6 ± 3.6	0.925
Frailty, *n* (%)	Pre-frail	72 (73.4%)	8 (25%)	<0.001
	Frail	26 (26.5%)	24 (75%)	
Preoperative MMSE		23 (21.8–25)	21.5 (20–22)	<0.001
Postoperative MMSE		23 (21–25)	22 (20–23.8)	0.075
**Preoperative morbidity, *n* (%)**				
Hypertension		57 (58.1%)	24 (24.7%)	0.081
Hyperlipidemia		8 (8.2%)	9 (6.9%)	0.004
Diabetes mellitus		11 (11.3%)	11 (8.5%)	0.003
Cerebrovascular disease		32 (33.0%)	19 (14.6%)	0.008
**Intraoperative doses of the vasoactive drugs (mg)**				
Norepinephrine		0.8 (0.41–1.28)	0.74 (0.3–1.29)	0.322
Dopamine		7.1 ± 3.1	7.7 ± 3.2	0.289
**Biochemical indices**				
Hemoglobin (g/L)		12.7 (11.6–14.1)	11.5 (8.9–12.6)	<0.001
White blood cell (×10^9^/L)		5.7 (4.5–7.4)	6.4 (4.7–8.7)	0.289
Albumin (g/L)		38.9 (35.7–42.7)	37.0 (32.4–39)	0.002
Serum creatinine (umol/L)		81.2 (70.5–97.9)	91 (70.5–124.3)	0.088
NT-proBNP (pg/ml)		711 (219.8–2,243)	1,474 (416.8–3,359)	0.055
cTnI (ng/ml)		1.4 (0.2–2.6)	0.8 (0.2–2.6)	0.97
CK-MB (ng/ml)		14.2 (2.6–20.7)	13.8 (2.4–22.2)	0.756
**S100β protein (μg/L)**				
T1		0.16 (0.09–0.24)	0.19 (0.14–0.25)	0.263
T9		0.22 (0.15–0.32)	0.37 (0.21–0.63)	0.072
T10		0.22 (0.12–0.31)	0.26 (0.17–0.40)	0.072
T11		0.23 (0.14–0.31)	0.23 (0.14–0.32)	0.918
T12		0.24 (0.14–0.35)	0.22 (0.14–0.30)	0.359
**The operation type, *n* (%)**				
Valve		58 (59.2%)	13 (40.6%)	0.151
CABG		13 (13.3%)	10 (31.3%)	
Aortic		7 (7.1%)	3 (9.4%)	
Combination		9 (9.2%)	4 (12.5%)	
Others		11 (11.2%)	2 (6.3%)	
**Echocardiography indices**				
LVEF (%)		57 (48–60)	52.5 (42–60.8)	0.272
LAd (mm)		43 (39–49.3)	43 (39.3–48)	0.994
LVd (mm)		53 (48–60.3)	55 (47.3–61.8)	0.489
**Time indices**				
Operation time (min)		192.5 (175–230)	262.5 (197.5–327.5)	<0.001
CPB time (min)		79 (70–105)	98.5 (68.8–124)	0.021
Aortic cross clamp time (min)		51 (40–75)	59 (42.3–89.8)	0.211
Mechanical ventilation time (h)		10 (7–15)	9.5 (8–14.8)	0.963
The length of stay in CICU (days)		4 (3–5.3)	4 (3–6.8)	0.679
The length of stay in hospital (days)		22.5 (19–27.3)	23 (18–30)	0.957

ASA, American Society of Anesthesiologists; NYHA, New York Heart Association; BMI, body mass index; MMSE, mini-mental state examination; cTnI, cardiac troponin I; NT-proBNP, N-terminal pro-B-type natriuretic peptide; CM-MB, creatine kinase-MB; CABG, coronary artery bypass grafting; CPB, cardio-pulmonary bypass.

### Tissue oxygen saturation

SctO_2_ (T_2_), and SctO_2lowest_ were found as significant risk factors for POD. The two groups showed no statistically significant difference in SrtO_2_ and SmtO_2_ (Table 2).

**Table 2:** Results of univariate logistic regression analysis of potential tissue oxygen saturation risk factors.

	Non-POD (*n* = 98)	POD (*n* = 32)	*P*-value
**SctO_2_**			
T0	65 (62–68)	64 (60–67.8)	0.05
T1	64.5 (62–70)	64 (58–68)	0.06
T2	68.4 ± 4.9	65.4 ± 5.2	0.004
T3	67 (64–73)	66.5 (62.3–70)	0.269
T4	65.3 ± 6.1	63.4 ± 6.2	0.137
T5	66.2 ± 5.9	65.3 ± 6.8	0.435
T6	64.5 (62–68)	64 (62–67)	0.296
T7	64 (61.8–68)	64 (61.3–66.8)	0.778
T8	66.5 (64–69.3)	64 (62–69.8)	0.169
T9	66 (64–70)	64 (61.3–67.8)	0.018
T10	66.5 (64–71.3)	65 (62–69.5)	0.147
T11	66 (63–70)	64.5 (62–68)	0.209
T12	65.5 (63.8–70)	66 (62.3–69.8)	0.981
SctO_2baseline_	65 (62.5–69)	63.5 (59–68)	0.05
SctO_2lowest_	59 (57–62)	55 (49.3–59)	<0.001
SctO_2decrease_	9.4 (6.2–13.9)	9.5 (5.3–22.4)	0.392
**SmtO_2_**			
T0	66.4 ± 10.4	67.5 ± 10.5	0.584
T1	64 ± 10.9	63.5 ± 10.2	0.798
T2	66.4 ± 10.4	67.5 ± 10.5	0.584
T3	63.7 ± 8.9	64 ± 8	0.849
T4	62 (53–68.3)	62 (55–66.5)	0.858
T5	62 (51.8–68)	61 (53.5–64)	0.49
T6	64.3 ± 10.8	61.8 ± 9.8	0.238
T7	64 (59–72.3)	61.5 (52.3–69)	0.206
T8	67 (62–72)	65.5 (60–69.8)	0.073
T9	64.6 ± 10.1	63.3 ± 8.5	0.49
T10	66.8 ± 10.1	63.2 ± 6.8	0.059
T11	66.1 ± 9	64.3 ± 8.3	0.306
T12	66.8 ± 8.8	65.3 ± 6.8	0.36
SmtO_2baseline_	65.2 ± 9.9	65.5 ± 9.7	0.881
SmtO_2lowest_	51.5 (48–59.3)	52 (49–57.8)	0.657
SmtO_2decrease_	14.5 (6.6–26.9)	17.5 (6.3–28.4)	0.865
**SrtO_2_**			
T0	69 (66.8–75)	68 (64.3–72)	0.268
T1	68.7 ± 6.5	67.9 ± 6.4	0.571
T2	69 (66.8–75)	68 (64.3–72)	0.268
T3	68 (65–72)	65.5 (64.3–70)	0.192
T4	66.5 (63.8–73)	67 (64–69.8)	0.647
T5	67 (63–73.5)	65 (62.5–70.8)	0.526
T6	67 (62–72)	65.5 (62–68)	0.245
T7	67 (61.8–72)	64.5 (62–69)	0.316
T8	69.1 ± 6.6	68.1 ± 4.2	0.426
T9	66.1 ± 5.4	66.7 ± 5.8	0.572
T10	67 (63–72)	67 (62–72)	0.598
T11	67 ± 6.1	67.7 ± 6.8	0.598
T12	66.9 ± 6.2	66.8 ± 6.1	0.972
SmtO_2baseline_	69.2 ± 5.6	68.2 ± 5.3	0.356
SmtO_2lowest_	60.5 (56–62)	59 (56.3–62)	0.374
SmtO_2decrease_	12.8 (6.8–18.0)	11.3 (8.3–16.9)	0.976

SctO_2_, cerebral oxygen saturation; SrtO_2_, renal oxygen saturation; SmtO_2_, brachioradialis oxygen saturation.

### Multivariate logistic regression analysis

Only the FI (OR 0.27, 95% CI 0.063–0.909), preoperative MMSE score (OR 0.71, 95% CI 0.528–0.954), and operation time (OR 1.012, 95% CI 1.002–1.022) remained significant in the multivariate analysis (*P* < 0.05) (Table 3).

**Table 3:** Results of multivariate logistic regression analysis.

	OR	95% CI		*P*-value
Frailty	0.27	0.063	0.909	0.036
Preoperative MMSE	0.71	0.528	0.954	0.023
Operation time	1.012	1.002	1.022	0.015

MMSE, mini-mental state examination.

**Figure 2:**
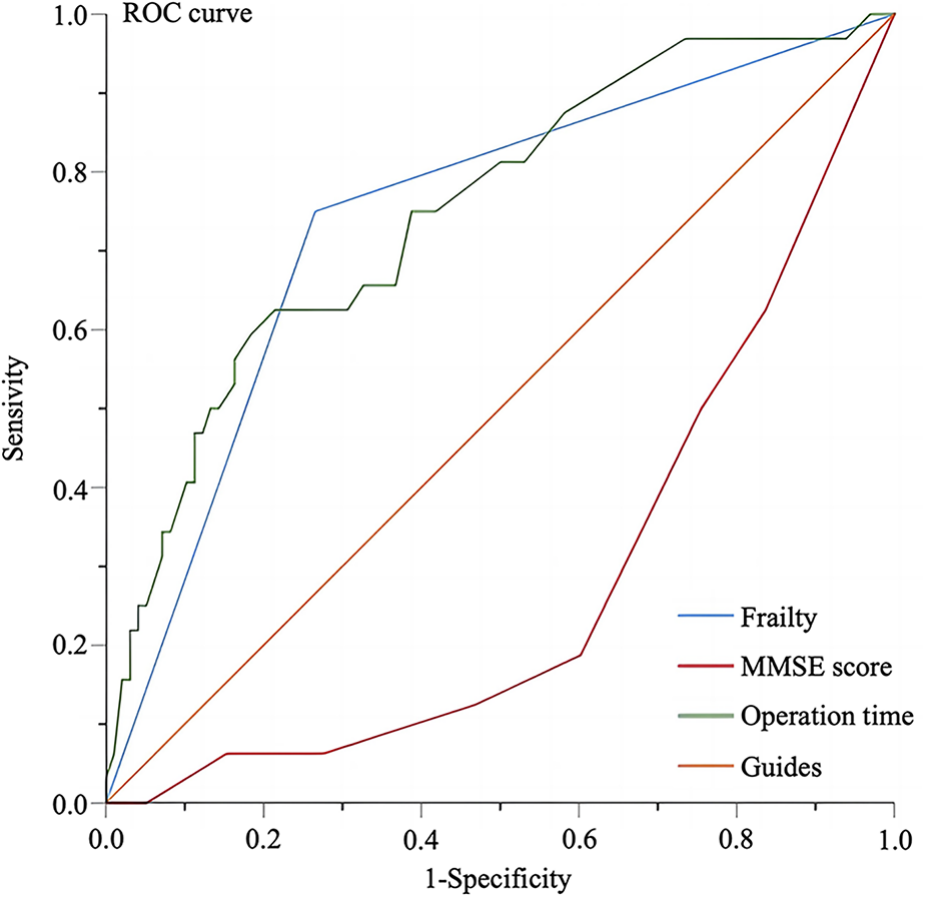
ROC curves on the predictive value of relevant factors for POD. ROC, receiver operating characteristic; MMSE, minimum mental state examination.

The FI (AUC 0.742, 95% CI 0.642–0.843, *P* < 0.001), preoperative MMSE score (AUC 0.281, 95% CI 0.184–0.378, *P* < 0.001), and operation time (AUC 0.751, 95% CI 0.652–0.849, *P* < 0.001) were found as significant predictive factors for POD (Figure 2). Both sensitivity and specificity were high for the FI (cut-off value, 0.27; sensitivity, 75%; specificity, 73.5%) and operation time (cut-off value, 237.5; sensitivity, 62.5%; specificity, 78.6%). However, while the sensitivity for the preoperative MMSE score was high, the specificity was found to be 0% (cut-off value, 19; sensitivity, 100%, specificity, 0%).

## Discussion

Among the elderly patients undergoing elective on-pump cardiac surgery, 50 (38.5%) were considered frail at baseline, and demonstrated a POD incidence of 46%. This is in contrast with the 80 (61.5%) pre-frailty patients, who demonstrated a POD incidence of 11.3%, which was significantly lower. The pathogenesis of frailty is multifactorial, and can be the result of chronic diseases, unhealthy lifestyles, and activity limitation. The reduced physiological reserve, coupled with the impaired resistance to surgical stress, thereby render a poor prognosis inevitable among frail patients (20, 21). In line with this, patients of the frailty group demonstrated greater comorbidities at baseline. Such patients further associated with lower cardiac function grades, which corroborated with the need for significantly higher doses of intraoperative dopamine compared to their counterparts. In addition, the rate of postoperative pulmonary complications was significantly higher among frail patients. Frail patients also reported longer length of hospital stay. Although statistically insignificant, this implied a longer recovery duration among such patients.

Frailty was demonstrated to significantly associate with POD in our study. Frailty was assessed in terms of FI, which employs a cumulative deficits approach, and has been considered an effective predictor of adverse outcome (22). As reported in the meta-analysis by Kojima et al., every 0.1 increase in FI was associated with a 28% increase in mortality risk (23). Importantly, our study found frailty as both an independent risk factor and a predictive factor for POD following cardiac surgery. In general, our findings fit with those of prior research. Although our study did not explore the association of frailty with postoperative mortality, both the operation time and postoperative pulmonary complication rate were higher in patients of the frailty group than those of the pre-frailty group, which may also be predictive of poor outcomes in patients with frailty.

The exposure of blood to foreign material during extracorporeal circulation may precipitate widespread inflammatory responses which can lead to postoperative neurocognitive decompensation (24). Hemodynamic fluctuations involved during CPB may further disrupt the perfusion of brain tissues and result in cerebral ischemia, one of the risk factors of POD (25). Conventional arterial pressure monitoring techniques such as MAP have been considered inadequate for the accurate reflection of cerebral perfusion (26, 27). Near-infrared spectroscopy (NIRS) is an emerging noninvasive brain imaging technology that is portable, mobile, and low-cost (28). It utilizes near-infrared light from 650 to 900 nm to determine cerebral oxygenation, blood flow, and metabolic status of localized regions of the brain. NIRS enables the continuous monitoring of rSO_2_, which allows for timely detection of cerebral hypoxia, and thereby plays an essential role in the protection against POD (29). In the observational study by Eertmans et al. (30) involving the continuous monitoring of rSO_2_ until 72 h after cardiac surgery, higher EuroSCORE II, lower preoperative MMSE score, and greater decrease in absolute postoperative rSO_2_ independently associated with POD. Our findings were partly in line with this, with frailty and the preoperative MMSE score identified as significant risk factors for POD, in addition to operation time. In contrast, SctO_2_ (T2) and SctO_2lowest_ did not demonstrate significant association to POD on multivariate analysis. This may be due to the small sample size of our study, which rendered insufficient statistical power, and the relatively short duration of monitoring. Renal oxygen saturation (SrtO_2_) has been often used to monitor the renal tissue blood perfusion to determine the presence of renal ischemia and hypoxia and to prevent the occurrence of acute renal injury (31). Simultaneously, other studies have described the monitoring the oxygen saturation of muscle tissue to assess the level of lactic acid in patients and thereby predict the body perfusion of patients (32). These results suggest that the oxygen saturation of renal and muscle tissue can reflect the blood perfusion level and oxygen-carrying capacity of the body to a certain extent. Our study show that the two groups showed no statistically significant difference in SrtO_2_ and SmtO_2_. SrtO_2_ and SmtO_2_ were not significant risk factors for POD.

Several studies have similarly identified operation time as a risk factor for cognitive dysfunction following surgery (33, 34). Prolonged duration of surgery not only increases the dosage of anesthetic drugs, but also increases the risk of cerebral ischemia and hypoxia (35). Our findings of an operation time of >237.5 min as an independent risk factor highlighted the importance of establishing a reasonable surgical plan to minimize the duration of surgery for the prevention of POD among frail elderly patients undergoing cardiac surgery.

There were several other limitations to this study. First, our results were limited by the single-center observational design. Second, as only cardiac surgeries involving extracorporeal circulation were considered, procedures such as off-pump coronary artery bypass grafting and aortic dissection were overlooked, thereby limiting the applicability of our results. Multi-center large-sample studies evaluating the association of frailty with POD following a wider range of cardiac surgeries are therefore warranted.

In summary, the FI, preoperative MMSE score, and operation time were independent risk factors for POD in elderly patients after cardiac surgery, with the FI and operation time showing a high predictive value, Cerebral oxygen saturation was associated with POD but was not an independent risk factor. Renal oxygen saturation and muscle oxygen saturation were not significant risk factors for POD.

## Data Availability

The datasets presented in this study can be found in online repositories. The names of the repository/repositories and accession number(s) can be found in the article/Supplementary Material.
